# Measuring capability wellbeing in adults at different stages of life for use in economic evaluation of health and care interventions: a qualitative investigation in people requiring kidney care

**DOI:** 10.1007/s11136-021-02851-z

**Published:** 2021-05-11

**Authors:** Paul Mark Mitchell, Samantha Husbands, Sabina Sanghera, Fergus John Caskey, Jemima Scott, Joanna Coast

**Affiliations:** 1 Health Economics Bristol (HEB), Population Health Sciences, Bristol Medical School, University of Bristol, Bristol, UK; 2 Population Health Sciences, Bristol Medical School, University of Bristol, Bristol, UK; 3 Southmead Hospital, North Bristol NHS Trust, Bristol, UK

**Keywords:** Health economics, Patient reported outcome measures, ICECAP-A, ICECAP-O, Life course

## Abstract

**Purpose:**

Capability wellbeing measures, such as the ICECAP measures, have been proposed for use in economic evaluations to capture broader outcomes of health and care interventions. The ICECAP measures have been developed to reflect capabilities at different stages of life. Some patient groups include patients of different ages and at different stages of life, so it is not always apparent which ICECAP measure is most relevant. This study explores the impact of age and life stage on completion, where both ICECAP-A and ICECAP-O were completed by the same patient.

**Methods:**

A think-aloud study, and an associated semi-structured interview were conducted with people receiving kidney care as a renal outpatient, kidney transplant outpatient, or through receiving facility-based haemodialysis. Qualitative analysis focused on (1) differences in responses across measures by individuals, where attributes had conceptual overlap, (2) key factors in self-reported capability levels, and (3) measure preference.

**Results:**

Thirty participants were included in the study, with a mix of older and younger adults. Attributes with similar wording across measures produced similar responses compared to attributes where wording differed. Age and health were key factors for self-reported capability levels. ICECAP-A was slightly preferred overall, including by older adults.

**Conclusion:**

This study suggests use of ICECAP-A in patients with certain chronic health conditions that include a mix of adults across the life course. This study highlights the importance of considering the stage of life when using capability measures and in economic evaluations of health and care interventions more generally.

**Supplementary Information:**

The online version contains supplementary material available at 10.1007/s11136-021-02851-z.

## Introduction

The use of patient reported outcome measures (PROMs) that aim to capture individuals’ capability wellbeing during health and care interventions has grown considerably over the last decade [1]. This trend is likely to continue now that regulatory bodies including the National Institute for Health and Care Excellence (NICE) in England and a similar group in the Netherlands (Zoorginstitut Nederland) have recommended that capability measures can be included in the economic evaluation of interventions under certain circumstances, such as in social care evaluations [2] or for long-term care [3]. Capability wellbeing measures attempt to capture people’s capabilities in terms of their ability to do and be the things in life that matter to them [4]. The ICECAP suite of measures provides one option for assessing capability wellbeing; it includes a measure for Adults (the ICECAP-A) [5], a measure specifically developed for Older people (the ICECAP-O) [6, 7] and a Supportive Care Measure for those nearing the end of life (ICECAP-SCM) [8].

Typically, PROMs used in economic evaluation of health and care interventions tend to focus exclusively on a person’s health-related quality of life [9]. For example, the EQ-5D instrument asks about a person’s health today and measures problems related to mobility, self-care, usual activities, pain/discomfort and anxiety/depression [10]. The EQ-5D is generic in terms of health dimensions designed for use across adults of all ages and at all stages of life. Although there are several alternative generic health measures [11], the EQ-5D is, internationally, the most commonly recommended PROM for economic evaluation of health technology assessments [12].

The ICECAP-A (Online Resource 1) was developed for all adults 18 and above whilst the ICECAP-O (Online Resource 2) was developed specifically for older people aged 65 and above. Both have similar measurement properties (see Table 1). Where decision-makers want to use an ICECAP measure (or measures), however, a key question is which ICECAP measure to use for patient groups that contain a mixture of younger and older adults, as both the ICECAP-A and ICECAP-O include capabilities relevant for older adults.

**Table 1:** Comparison of wording of similar attributes on ICECAP-A and ICECAP-O

Comment	ICECAP-A	ICECAP-O
1	STABILITY	SECURITY
Description	Feeling settled and secure	Thinking about the future without concern
Wording	worded differently	
2	ATTACHMENT	ATTACHMENT
Description	Love, friendship and support	Love and friendship
Wording	similar	extra "…*that I want*" at end of each level (O)
Top level (4)	*a lot*	*all*
level 3	*quite a lot*	*a lot*
3	AUTONOMY	CONTROL
Description	Being Independent	Independence
Wording	the same, bar description	
4	ACHIEVEMENT	ROLE
Description	Achievement and progress	Doing things that make you feel valued
Wording	worded differently	
5	ENJOYMENT	ENJOYMENT
Description	Enjoyment and pleasure	Enjoyment and pleasure
Wording	similar	extra"…*that I want*" at end of each level (O)
Top level (4)	*a lot*	*all*
level 3	*quite a lot*	*a lot*

Comparison of ICECAP-A and ICECAP-O attributes ordered by ICECAP-A attributes

Work has recently suggested that what people value and how much it is valued may vary throughout the life course, and that measures included in economic evaluation should account for this [13]. Evidence derived from the development of the ICECAP measures suggests that capability attributes differ at different points in the life course [13]; qualitative research has suggested that some older people find some attributes of ICECAP-A difficult to complete due to the relevance of attributes (i.e. achievement) in later life [14] and that those very close to death find the ICECAP-SCM attributes most meaningful compared to EQ-5D and ICECAP-A [15]. More specific evidence is required, however, about which capability measures might be most appropriate in particular contexts. There is no existing evidence, with most studies to date focusing instead on exploring one ICECAP measure alongside other health-related measures for different patient groups [16, 17].

This study aims to explore the use of both ICECAP-A and ICECAP-O amongst patient groups where either measure could be used. It focuses on people who have a chronic health condition which often, but not always, has an onset later in life: people requiring kidney care but undergoing a variety of interventions. The median age at the point of commencing renal replacement therapy is 63.7 years of age in the UK [18]. When patients are grouped around this age, it is not clear which ICECAP measure to use, the ICECAP-A or ICECAP-O.

## Methods

### Measures: ICECAP-A and ICECAP-O

**Fig. 1:**
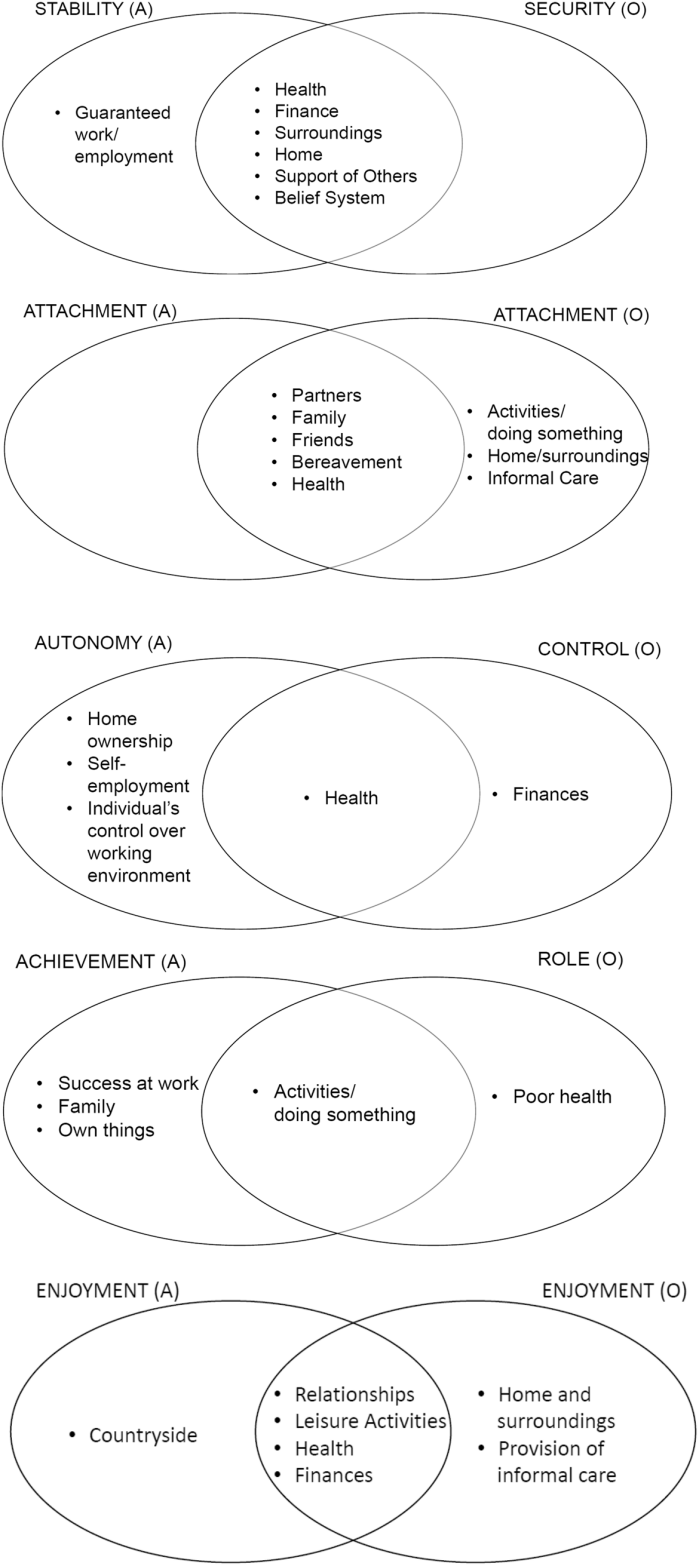
Attribute conceptual overlap between ICECAP-A and ICECAP-O, based on reporting from the original ICECAP development papers [5, 6]

Three of the attributes across ICECAP-A and ICECAP-O are worded somewhat comparably; these are related to a person’s capabilities to have attachment, enjoyment and autonomy/control (expressed in both cases in terms of independence—see Table 1). Although the two remaining attributes differ conceptually across the two measures, the concepts overlap; these are related to a person’s capability to have (i) achievement (ICECAP-A) or a role (ICECAP-O) and (ii) stability (ICECAP-A) or security (ICECAP-O) in their lives [13]. The ICECAP-A and ICECAP-O were developed at different times and for two different groups (all adults and older people). Each was developed through qualitative interviews in two stages with the relevant group. The generation of conceptual attributes and the development of meaningful wording thus happened independently in the two studies. Figure 1 demonstrates the conceptual overlap in the five comparable attributes for the ICECAP-A and ICECAP-O alongside the various influences on quality of life reported for each attribute in the measure development papers for the respective ICECAP measures [5, 6].

**Fig. 2:**
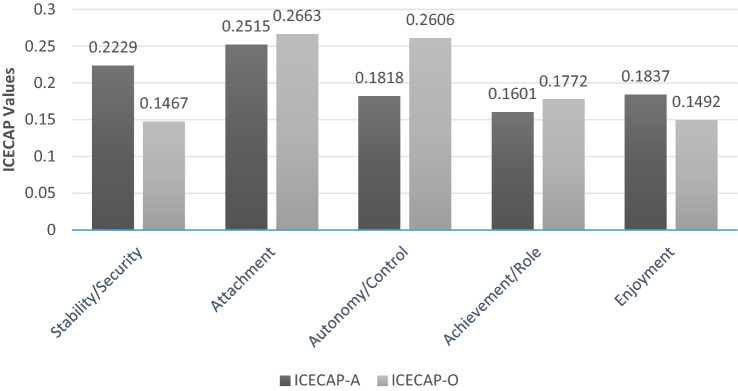
Overall contribution to score for ICECAP-A and ICECAP-O attributes from UK population samples (Data from Coast et al. [7] and Flynn et al. [19]. Difference between the top and bottom level of each attribute)

Choice of ICECAP measure is also important in terms of the weight that is given to each attribute. Figure 2 illustrate the relative importance placed on different capability attributes using the existing ICECAP population valuation surveys available; for the ICECAP-A, the values were from a sample of all adults [19]; for the ICECAP-O, the values are from a sample of older adults [7]. For example, the over 65 UK population appear to value the capability for control relative to the other ICECAP-O attributes, more than the general adult population (including older adults) values autonomy relative to the other ICECAP-A attributes.

### Study design

A think-aloud study, whereby participants complete a task whilst thinking out loud [20], was conducted between April and July 2017 for people requiring kidney care. Think-aloud interviews have been shown to generate more insightful information concerning the response processes when completing a task compared to retrospective methods [21]. Ethics approval was obtained from the East of England NHS Research Ethics Committee (16/EE/0331). Further details on the study design, as well as the response process validity of the three measures, are available in Online Resource 3 and elsewhere [22].

### Sampling

Participants were recruited from lists of patients who were booked to attend a large secondary care centre in the UK and were receiving either monitoring of chronic kidney disease or kidney transplantation through the renal outpatient clinic, or were receiving facility-based haemodialysis. Purposeful sampling was used to achieve diversity in age and type of kidney care received [23].

### Data collection

Participants were asked to think-aloud whilst completing ICECAP-A, ICECAP-O and EQ-5D-5L [24]. Order of completion of ICECAP-A and ICECAP-O alternated between first and third to ensure each measure appeared first on a similar number of instances and that these two measures were not completed consecutively, given their similarity. A semi-structured interview was conducted following the think-aloud task to clarify issues arising in the task and to learn about participants’ views about the measures they completed. All interviews were audio-recorded and fully transcribed.

### Analysis

Think-aloud interview data were initially analysed by reading and re-reading transcripts. Codes were developed both inductively (PM, JC) based on the analysis of five transcripts, and deductively, based on the factors influencing each of the capability attributes, as described in the development papers for ICECAP-A [5] and ICECAP-O [6] (see Fig. 1). A coding framework was developed from this and applied to all transcripts.

Data from all transcripts were then analysed using the framework method [25] and linking with relevant theory [26] in terms of capabilities—that is the freedom of people to do and be the things in life that are important to them [4, 27]. The framework method facilitated the comparison of responses between measures and participants.

The primary analysis focuses on the qualitative data provided in the think-aloud completion of ICECAP measures, as well as the semi-structured interview that followed. ICECAP-A [19], ICECAP-O [7] and EQ-5D-5L UK cross-walk [28] value sets were used to produce summary scores. The EQ-Visual Analogue Scale (EQ-VAS), that has a range from 0 to 100 worst possible health—best possible health imaginable scale, is also reported. Within person differences between ICECAP scores are also reported (i.e. difference between ICECAP-A and ICECAP-O scores for each individual).

#### Analysis of measure responses

The first part of the analysis focuses on the responses that participants gave to the comparable attributes of ICECAP-A and ICECAP-O (see Table 1 and Fig. 1). For both ICECAP-A and ICECAP-O, each measure has 5 attributes, with four response levels per attribute ranging from full capability (4) to no capability (1) in that given attribute. If we assume that overlapping attributes across ICECAP measures are capturing similar capability, in most instances this is a like for like response level comparison (e.g. a level 1 response on the stability attribute for ICECAP-A would expect a level 1 response on the security attribute on ICECAP-O). The only difference is that wording for level 3 on ICECAP-O (“a lot” of capability) is most similar to the wording for level 4 on ICECAP-A for the attachment and enjoyment attributes, so either a level 3 or level 4 response on ICECAP-O would match the top level on ICECAP-A in these instances (see Online Resource 4). Descriptive analysis explores how comparable responses are across measures. Where this varied, qualitative data were analysed to explore reasons for differences.

#### Analysis of qualitative data

Framework analysis [25] of the think-aloud responses was conducted to see what factors participants were drawing upon to provide their current capability levels on ICECAP-A and ICECAP-O, based on the areas covered in Fig. 1. Each area identified in the five Venn diagrams in Fig. 1 was given a column within a matrix to see when and in what circumstances each was verbally reported by participants when answering.

To explore the influence of age and preferences between ICECAP measures, the sample was split in two so that one sample consisted of the intended age range for ICECAP-O (i.e. 65 and above [7]) and the second consisted of adults younger than age 65. Themes relating to age in completing the ICECAP measures were extracted from the think-aloud data. Preferences for differences between measures were drawn from the semi-structured component of the interview where a direct question asked participants about what ICECAP measure they preferred.

The results section is structured to first analyse measure responses and factors influencing capability levels across the whole sample. The final part of the results section splits the sample in two by age to look at preferences between measures across older and younger adults.

## Results

**Fig. 3:**
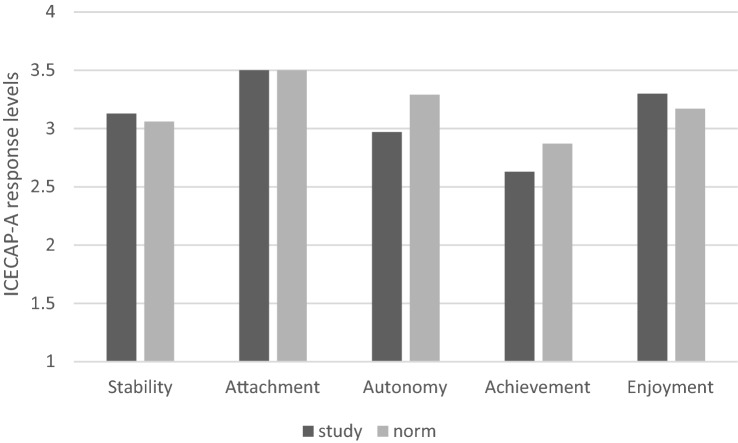
ICECAP-A study response levels compared to population norm data (norm data from [29])

**Fig. 4:**
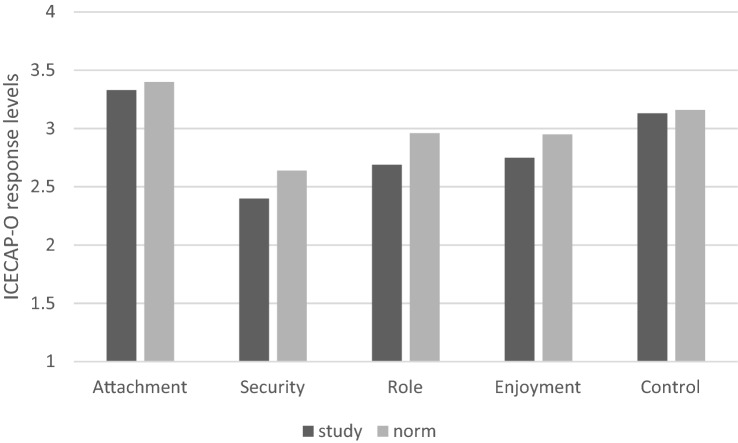
ICECAP-O study response levels compared to population norm data (norm data from [30])

Table 2 presents descriptive statistics for the sample. In total, 30 participants were included in the think-aloud and semi-structured interview analysis, with 12 of those participants aged 65 and over and 18 under 65 years of age. Older participants tended to have slightly lower mean capability on both measures: ICECAP-A (0.80 versus 0.82) and ICECAP-O (0.78 versus 0.80). Focusing on within person differences across measures, the absolute mean difference in ICECAP-A and ICECAP-O scores for each participant was 0.07 (standard deviation 0.06), with larger differences in reporting between the older participants (0.08) compared to the younger adults (0.06). Average response levels for ICECAP-A and ICECAP-O attributes are reported in Fig. 3 and 4 and compared to ‘norm’ population data for ICECAP-A in 2010 [29] and ICECAP-O in 2005–2006 [30]. Average shortfall in capability attributes compared to the ‘norm’ data are highest in autonomy (0.34 reduction) followed by achievement (0.24) on the ICECAP-A. For the ICECAP-O, highest average shortfall is similar for three attributes: role (0.27), security (0.24), and enjoyment (0.20).

**Table 2:** Sample descriptive statistics

Sex		Age	
Male	23	75+	4
Female	7	65–74	8
Ethnicity		55–64	7
White	28	45–54	6
Non-white	2	35–44	4
		18–34	1
Outcome scores (s.d.)			
EQ-5D-5L	0.61 (0.27)	Kidney care received	
EQ-5D-VAS	67 (18)	Kidney outpatients	18
ICECAP-A	0.81 (0.17)	Transplant outpatients	6
ICECAP-O	0.79 (0.16)	Facility Haemodialysis	6

### Analysis of measure responses

Unsurprisingly, attributes that differ somewhat in concept, and therefore most in wording (i.e. Stability-Security and Achievement-Role) varied the most from predicted responses (see Table 3). Responses were broadly as expected across the three attributes worded comparably across ICECAP-A and ICECAP-O (i.e. attachment, autonomy-control and enjoyment).

**Table 3:** Comparison of similar attribute responses across ICECAP-A and ICECAP-O

	Stability-Security	Attachment (A-O)	Autonomy-Control	Achievement-Role	Enjoyment (A-O)
Predicted*	11/30	28/30	22/30	18/29	22/30
1 level variation	12/19	2/2	7/8	10/11	6/8
2 level variation	5/19	0/0	1/8	1/11	2/8
3 level variation	2/19	0/0	0/0	0/0	0/0
Higher capability levels	17/19 (A)	6/30 (A)	6/8 (O)	6/11 (O)	8/8 (A)

*Predicted response levels across ICECAP-A and ICECAP-O—see Online Resource 4 for predictions

The least comparable responses across attributes were for the stability attribute on ICECAP-A and the security attribute on ICECAP-O with 11 out of 30 responses as predicted (see Online Resource 4). Seventeen of the 19 participants with varying response levels recorded higher stability levels on ICECAP-A compared to security levels on ICECAP-O, with six having two or more response level variation than what was anticipated. The question on security on ICECAP-O appeared to focus participants’ minds on the impact that their current health state was having on them and people close to them, whereas participants seemed to answer the stability attribute (ICECAP-A) more broadly, considering things other than health such as financial security:

Settled and secure. Difficult. Certainly not unable. It’s got to be more than a few. Financially safe. I think it must be the top one

(Participant 7, male, age 65-74, ICECAP-A stability level 4)

And as I’ve said just now…my kidneys are failing…I think about it a lot

(Participant 7, male, age 65-74, ICECAP-O security level 1)

Seventeen responses across the achievement (ICECAP-A) and role (ICECAP-O) attributes were as expected (see Online Resource 4). Age appeared to play a role in cases where unexpected responses occurred, with most differences amongst older participants, as demonstrated by the following participant:

Well at my age I don’t think I can achieve and progress in aspects of my life really. I’ve done what I could and I’m happy with what I’ve done

(Participant 4, female, aged 75+, ICECAP-A achievement level 1)

Am I able to do most things? No. Am I able to do things that make me feel valued? Yes

(Participant 4, female, aged 75+, ICECAP-O role level 3)

### Analysis of key factors in self-reported capability levels

Tables 4 and 5 report the spontaneously reported factors influencing participants’ responses during the think-aloud interview, as elicited from the ICECAP measures’ development papers (see Fig. 1).

**Table 4:** Spontaneously reported factors* for ICECAP-A responses (*n* = 30)

Stability	Attachment	Autonomy	Achievement	Enjoyment
Support for others (13)	Family (13)	Health (15)	Activities/doing something (8)	Relationships (8)
Health (9)	Friends (12)	Control over their working environment (1)	Family (4)	Health (7)
Home (7)	Partners (9)	Home ownership (0)	Success at work (4)	Leisure activities (6)
Finance(4)	Health (3)	Self-employment (0)	Own things (0)	Finances (2)
Guaranteed work/employment (2)	Bereavement (0)			Countryside (0)
Belief system (0)				

*Factors related to areas aimed to be covered by ICECAP-A attributes (see Fig. 1)

**Table 5:** Spontaneously reported factors for ICECAP-O responses (*n* = 30)

Security	Attachment	Control	Role	Enjoyment
Health (17)	Family (13)	Health (14)	Activities/doing something (17)	Leisure activities (12)
Support of others (8)	Friends (13)	Finances (3)	Health (7)	Health (10)
Finance (6)	Partners (9)			Relationships (4)
Home (1)	Home/surroundings (1)			Finances (1)
Belief system (0)	Health (0)			Home and surroundings (1)
	Bereavement (0)			Provision of informal care (1)
	Activities/doing something (0)			
	Informal carers (0)			

*Factors related to areas aimed to be covered by ICECAP-O attributes (see Fig. 1)

With the exception of the attributes for attachment, health is consistently reported as being an influence on all the capability attributes for ICECAP-A and ICECAP-O across both measures:

If these people weren’t here and didn’t have these techniques and this machine I’d be in a worse state you know, because my kidney would, it’d be organ failure.

(Participant 28, male, age 65–74, ICECAP-O security level 3 (ICECAP-A stability level 4))

but I guess without the medical help I probably wouldn’t be very independent

(Participant 16, male, aged 55–64, ICECAP-A autonomy level 3 (ICECAP-O control level 3))

Yeah the only thing that makes me feel bad is my work and I do that, no problem, obviously before when I was on dialysis it was a problem and now I’ve had a transplant it’s not

(Participant 23, male, aged 45–54, ICECAP-O role level 4 (ICECAP-A achievement level 4))

Health was also discussed by some for the achievement attribute on ICECAP-A, even though this is not listed as an influence in Fig. 1. A number of respondents made a clear link between their ability to achieve in areas of life that were important to them and their health state:

I could never work full-time when I was younger, so that held me back

(Participant 2, female, aged 45–54, ICECAP-A achievement level 3 (ICECAP-O role level 3))

Other common factors spontaneously mentioned during the think-aloud element of the interview included the ability to undertake meaningful activities (achievement (ICECAP-A), enjoyment (ICECAP-A and ICECAP-O), role (ICECAP-O)) and have relationships (attachment (ICECAP-A and ICECAP-O), enjoyment (ICECAP-A)), as well as support (stability ICECAP-A) and concern for others (security ICECAP-O):

Because I was so active previously, and so sort of hands on, I miss being able to do all those things now, and I feel very low about that, but within myself it is very frustrating, I cannot do the things I used to do.

(Participant 11, male, aged 55–64, ICECAP-O role level 2 (ICECAP-A achievement level 3))

I would say I am able to feel settled and secure in all areas of my life, as long as my carer’s with me

(Participant 3, male, aged 55–64, ICECAP-A stability level 4 (ICECAP-O security level 1))

obviously I think about the future, with concern, because of my age and I worry about my family, especially my daughter.

(Participant 4, female, aged 75+, ICECAP-O security level 2 (ICECAP-A stability level 3))

The factors influencing the responses to capability measures did appear to differ across the older and younger age groups, particularly for ICECAP-A attributes, suggesting that what these participants value has changed as they have aged. Examples of evidence where age appeared to play a role in how participants thought about and responded to attributes across both measures are illustrated below:

You would say personally I’m very independent, although increasingly as I get older I tend to seek companionship more than I did when I was younger, so I’m going to put I’m independent in many things.

(Participant 5, female, aged 45–54, ICECAP-O control level 3 (ICECAP-A autonomy level 3))

I’m 51 now and I think any progress and achievement I would’ve achieved by now, so I think my prospects are more limited than in the past so I’ve discounted that (highest capability level)

(Participant 5, female, aged 45–54, ICECAP-A achievement level 3 (ICECAP-O role level 3))

I care less about what people think about me than when I did when I was younger.

(Participant 1, male, aged 45–54, ICECAP-A enjoyment level 3 (ICECAP-O enjoyment level 3))

### Measure preference analysis

Of the 12 respondents aged 65 and older, seven preferred the ICECAP-A due to ease of completion and the importance they attached to the stability and attachment attributes on ICECAP-A.

I think what jumped out at me was feeling settled and secure. That sums up my life, I think, in a lot of ways…followed closely by love, friendship and support.(Participant 10, male, aged 65–74)

Four of the 12 older participants preferred the ICECAP-O due to the importance they placed on the security attribute on ICECAP-O, greater perceived depth than the ICECAP-A, more age appropriate and more clearly worded compared to the ICECAP-A.

Really, I thought the one talking about, thinking about the future, is very important, because I mean at my age that’s all you do

(Participant 4, female, aged 75+)

Of the 18 participants aged between 18 and 64, six preferred the ICECAP-A for its ease of completion, liking the achievement attribute on the ICECAP-A and not liking the role question on the ICECAP-O.

that’s (achievement on ICECAP-A) the only question out of all of it that made me really think about kind of my life going forward with where I am and the condition is never gonna go away.

(Participant 18, male, aged 35–44)

I am able to do all the things that make me feel valued. Bit of a funny question (role on ICECAP-O). Do all the things that make me feel valued… That is a bit vague that one.

(Participant 17, female, aged 35–44)

Six of the 18 in this group preferred the ICECAP-O for a perception of greater depth in what is covered compared to the ICECAP-A, liking the security attribute on ICECAP-O with its future focus and not liking the achievement question on the ICECAP-A.

Because that one gives you more of a general overview of the situation. These (ICECAP-O attributes), I think are going a lot deeper than those. (Participant 13, male, aged 55–64)

Six of the eighteen did not express a preference for either the ICECAP measures.

It’s just a lot of them were very, very similar…They’re all ok as far as I’m concerned with it (Participant 30, male, aged 55–64)

## Discussion

This study presents the first direct comparison of ICECAP-A and ICECAP-O responses by the same population with a chronic health condition. It is also the first exploration of how informants use different information to provide a response to two capability wellbeing measures. Attributes across ICECAP-A and ICECAP-O measures that were similarly worded were more likely to be completed as expected if attributes were aimed at capturing overlapping content. Nonetheless, the average difference in absolute mean capability scores across measures within participants is 0.07; this difference is comparable to capability levels found in a community survey between those who are and are not housebound for the ICECAP-O [31]. This suggests choice of measure should be carefully considered, given the difference in capability scores across measures could influence if health and care interventions are cost-effective when used in economic evaluations. ICECAP-A tended to lead to higher capability response levels for participants. For those who expressed a preference, ICECAP-A was preferred by marginally more in this sample, including amongst older adults, but there is no clear preference for one measure over the other. Age appeared to influence how participants responded across the two measures (see Sect. “Analysis of key factors in self-reported capability levels”), suggesting there could be a role for a life course approach [13], though age may not be the sole determinant of where a person sees themselves on the life course. Employment and retirement may be other important factors to consider in patient groups that include both. Although health was frequently cited as an influence on capability when completing either ICECAP-A or ICECAP-O, it is unclear to what extent that relates to the recruitment of participants as patients and the conduct of the majority of interviews within a health setting.

Only one previous study has used two ICECAP measures simultaneously (ICECAP-A and ICECAP-SCM) in patients requiring end of life care [15]. This earlier study suggested that patients closer to the end of life were more likely to find the ICECAP-SCM measure relevant, whilst those earlier in the trajectory towards death were more likely to favour ICECAP-A. The study reported here is the first to compare the use of ICECAP-A and ICECAP-O measures in a patient group where either instrument could be used. Although the research was conducted in one group of people requiring care, a range of participants were included in terms of age and treatment options. A limitation of the work is that, although the research focused on exploring different ICECAP measures for different forms of kidney care, issues of life stage were not explicitly explored with participants during interviews. For example, we do not know exactly who has a job and who is retired in this study. A further limitation is that most interviews were undertaken at a health facility in this study and this may have influenced participants thinking about their health when completing the ICECAP-A and ICECAP-O.

Health plays a prominent role in this study, and the finding that it is important in determining responses to most of the ICECAP-A (see Table 4) and ICECAP-O (see Table 5) attributes in this sample supports the ICECAP-A [5] and ICECAP-O [6] developers’ objectives of capturing health as an important influence on many of the attributes (see Fig. 1). This is apparent even though the word “health” does not appear in the attributes on either ICECAP measure investigated here. The findings from this study may be generalisable to other chronic health care settings, where the condition has similar characteristics to those associated with kidney disease. It would be worthwhile exploring whether health appears to be of a similar level of importance when the ICECAP measures are completed in non-health care settings.

This paper attempts to help policymakers and researchers alike in choosing ICECAP measure(s) for studies where patient groups straddle both the typical ages of employment and retirement for use in economic evaluation. There was only a slight preference for the ICECAP-A over ICECAP-O in this sample. Previous think-aloud analysis of these data suggests the ICECAP-A produces the fewest errors in completion, although the difference between the ICECAP-A and ICECAP-O is again marginal [22]. If reliability and other forms of validity in specific patient groups are considered more important [32], then there is currently only existing evidence on the construct validity in patients receiving dialysis or conservative care for the ICECAP-O [33].

A principal consideration, however, is likely to concern the use of ICECAP measures in health and care decision-making. Although current research is ongoing to develop an economic evaluation framework that would allow for the multiple use of existing ICECAP measures over the life course [13], the existing economic evaluation framework applied in health and care faces similar methodological questions when using a single outcome measure across all stages of the life course [34, 35]. With further evidence emerging on using ICECAP-A in decision-making [36], the short-term advice would appear to be in favour of using the ICECAP-A where there are adults across both age groups. Where there are mainly older adults, this choice might not be as straightforward given the potential difficulty for retired adults with the achievement attribute on ICECAP-A that has been highlighted elsewhere [14] and previous evidence in favour of using ICECAP-O in older adults compared to other PROMs [37]. There is, however, some evidence in this study that what matters to people in terms of their capability wellbeing does shift as they age and that ICECAP measure selection should be cognisant of the age range and possibly other life course factors of the patient sample under consideration.

## Supplementary Information

Below is the link to the electronic supplementary material.

Supplementary file1 (PDF 55 kb)

Supplementary file2 (PDF 20 kb)

Supplementary file3 (DOCX 24 kb)

Supplementary file4 (DOCX 17 kb)
