# Supplementary material for: Measuring capability wellbeing in adults at different stages of life for use in economic evaluation of health and care interventions: a qualitative investigation in people requiring kidney care
Source: Qual Life Res. 2021 May 11;30(10):2863–73. doi: 10.1007/s11136-021-02851-z (PMC8481176; doi:10.1007/s11136-021-02851-z)
Supplement: Supplementary file 1 — Supplementary file1 (PDF 55 kb) [file 11136_2021_2851_MOESM1_ESM.pdf]

ABOUT YOUR OVERALL QUALITY OF LIFE

Please indicate which statements best describe your overall quality of life at the moment by placing a tick (✓) in **ONE** box for each of the five groups below.

1. Feeling settled and secure

Statement	Box	Score
I am able to feel settled and secure in **all** areas of my life	☐	4
I am able to feel settled and secure in **many** areas of my life	☐	3
I am able to feel settled and secure in **a few** areas of my life	☐	2
I am **unable** to feel settled and secure in **any** areas of my life	☐	1

2. Love, friendship and support

Statement	Box	Score
I can have **a lot** of love, friendship and support	☐	4
I can have **quite a lot** of love, friendship and support	☐	3
I can have **a little** love, friendship and support	☐	2
I **cannot** have **any** love, friendship and support	☐	1

3. Being independent

Statement	Box	Score
I am able to be **completely** independent	☐	4
I am able to be independent in **many** things	☐	3
I am able to be independent in **a few** things	☐	2
I am **unable** to be at all independent	☐	1

4. Achievement and progress

Statement	Box	Score
I can achieve and progress in **all** aspects of my life	☐	4
I can achieve and progress in **many** aspects of my life	☐	3
I can achieve and progress in **a few** aspects of my life	☐	2
I **cannot** achieve and progress in **any** aspects of my life	☐	1

5. Enjoyment and pleasure

Statement	Box	Score
I can have **a lot** of enjoyment and pleasure	☐	4
I can have **quite a lot** of enjoyment and pleasure	☐	3
I can have **a little** enjoyment and pleasure	☐	2
I **cannot** have **any** enjoyment and pleasure	☐	1

Please ensure you have only ticked **ONE** box for each of the five groups.

© 2010 Hareth Al-Janabi and Joanna Coast
