# Supplementary material for: Measuring capability wellbeing in adults at different stages of life for use in economic evaluation of health and care interventions: a qualitative investigation in people requiring kidney care
Source: Qual Life Res. 2021 May 11;30(10):2863–73. doi: 10.1007/s11136-021-02851-z (PMC8481176; doi:10.1007/s11136-021-02851-z)
Supplement: Supplementary file 2 — Supplementary file2 (PDF 20 kb) [file 11136_2021_2851_MOESM2_ESM.pdf]

ABOUT YOUR QUALITY OF LIFE

By placing a tick (✓) in ONE box in EACH group below, please indicate which statement best describes your quality of life at the moment.

1. Love and Friendship

Statement		Score
I can have all of the love and friendship that I want	☐	4
I can have a lot of the love and friendship that I want	☐	3
I can have a little of the love and friendship that I want	☐	2
I cannot have any of the love and friendship that I want	☐	1

2. Thinking about the future

Statement		Score
I can think about the future without any concern	☐	4
I can think about the future with only a little concern	☐	3
I can only think about the future with some concern	☐	2
I can only think about the future with a lot of concern	☐	1

3. Doing things that make you feel valued

Statement		Score
I am able to do all of the things that make me feel valued	☐	4
I am able to do many of the things that make me feel valued	☐	3
I am able to do a few of the things that make me feel valued	☐	2
I am unable to do any of the things that make me feel valued	☐	1

4. Enjoyment and pleasure

Statement		Score
I can have all of the enjoyment and pleasure that I want	☐	4
I can have a lot of the enjoyment and pleasure that I want	☐	3
I can have a little of the enjoyment and pleasure that I want	☐	2
I cannot have any of the enjoyment and pleasure that I want	☐	1

5. Independence

Statement		Score
I am able to be completely independent	☐	4
I am able to be independent in many things	☐	3
I am able to be independent in a few things	☐	2
I am unable to be at all independent	☐	1

Tick one box only in each section

© Joanna Coast & Terry Flynn
