# Supplementary material for: Measuring capability wellbeing in adults at different stages of life for use in economic evaluation of health and care interventions: a qualitative investigation in people requiring kidney care
Source: Qual Life Res. 2021 May 11;30(10):2863–73. doi: 10.1007/s11136-021-02851-z (PMC8481176; doi:10.1007/s11136-021-02851-z)
Supplement: Supplementary file 3 — Supplementary file3 (DOCX 24 kb) [file 11136_2021_2851_MOESM3_ESM.docx]

**Online Resource 3 from *Quality of Life Research* publication:**

**Measuring capability wellbeing in adults at different stages of life for use in economic evaluation of health and care interventions: a qualitative investigation in people requiring kidney care**

**Paul Mark Mitchell, Samantha Husbands, Sabina Sanghera, Fergus John Caskey, Jemima Scott, Joanna Coast**

**Corresponding author:** Paul Mark Mitchell ([paul.mitchell@bristol.ac.uk](mailto:paul.mitchell@bristol.ac.uk)), Health Economics Bristol, 1-5 Whiteladies Road, Population Health Sciences, Bristol Medical School, University of Bristol, UK, BS8 1NU.

**Further details of sampling and data collection**

This research used a ‘think-aloud’ method with a subsequent semi-structured interview. During the think-aloud, individuals were asked to verbalise their thought process whilst they completed three measures [1]. This process avoids interrupting the task completion. It enables examination of the problems that respondents encounter in terms of their comprehension of the measure, their ability to retrieve relevant information, make a judgement and come to an appropriate response. During the think-aloud part of the interview, the interviewer remains silent, so long as individuals continue to think-aloud [2].

**Sampling and recruitment**

Samples for previous think-aloud studies on health and capability patient reported outcome measures have ranged from 10 [3] to 34 [4]. Here, it was expected that a sample size of at least 25 patients would be enough to enable the analysis of the think-aloud tasks, and to explore the use of the three measures (EQ-5D-5L, ICECAP-A and ICECAP-O) for people requiring kidney care.

Patients were recruited through the outpatient units of a large UK secondary care renal centre. Included individuals had chronic kidney disease, were willing and able to provide informed consent to participate, and were able to communicate in English (required given that the study was exploring an English language questionnaires). Patients were further sampled purposefully to achieve diversity in age (<65 or >65) and type of kidney care received. Patients were initially approached by mail, including a study information sheet, and then telephoned to determine whether they were willing to participate in the study and to obtain consent.

**Data collection**

Interviews were conducted in the patient’s home or in the renal centre by PM. Questions began by obtaining socio-demographic information. Participants completed two warm-up tasks to introduce the idea of thinking-aloud and were then asked to think-aloud whilst completing the three questionnaires. Completion of ICECAP-A and ICECAP-O was always separated by completion of EQ-5D-5L because of the similarities between the two ICECAP measures. The researcher remained silent whilst participants completed the measure and thought-aloud; the researcher only spoke to remind participants to keep thinking aloud if they were silent for more than 10 seconds. The subsequent semi-structured interview explored perceptions of the measures, as well as probing issues arising from the think aloud task. Interviews were audio recorded and transcribed verbatim. Interview and questionnaire data were managed in Microsoft Word and Excel.

**References**

1. Willis, G. B. (2004). Cognitive interviewing: A tool for improving questionnaire design: Sage publications.

2. Kuusela, H., & Pallab, P. (2000). A comparison of concurrent and retrospective verbal protocol analysis. The American journal of psychology, 113(3), 387.

3. van Leeuwen, K. M., Jansen, A. P. D., Muntinga, M. E., Bosmans, J. E., Westerman, M. J., van Tulder, M. W., & van der Horst, H. E. J. B. H. S. R. (2015). Exploration of the content validity and feasibility of the EQ-5D-3L, ICECAP-O and ASCOT in older adults. BMC Health Services Research, 15(1), 201.

4. Al-Janabi, H., Keeley, T., Mitchell, P. M., & Coast, J. (2013). Can capabilities be self-reported? A think aloud study. Social Science & Medicine, 87, 116-122.
