# Supplementary material for: Measuring capability wellbeing in adults at different stages of life for use in economic evaluation of health and care interventions: a qualitative investigation in people requiring kidney care
Source: Qual Life Res. 2021 May 11;30(10):2863–73. doi: 10.1007/s11136-021-02851-z (PMC8481176; doi:10.1007/s11136-021-02851-z)
Supplement: Supplementary file 4 — Supplementary file4 (DOCX 17 kb) [file 11136_2021_2851_MOESM4_ESM.docx]

**Online Resource 4 from *Quality of Life Research* Publication:**

**Measuring capability wellbeing in adults at different stages of life for use in economic evaluation of health and care interventions: a qualitative investigation in people requiring kidney care**

**Paul Mark Mitchell, Samantha Husbands, Sabina Sanghera, Fergus John Caskey, Jemima Scott, Joanna Coast**

**Corresponding author:** Paul Mark Mitchell ([paul.mitchell@bristol.ac.uk](mailto:paul.mitchell@bristol.ac.uk)), Health Economics Bristol, 1-5 Whiteladies Road, Population Health Sciences, Bristol Medical School, University of Bristol, UK, BS8 1NU.

**Table A1. Expected level comparability across ICECAP attributes**

| **ICECAP-A** **→** **ICECAP-O** | | **ICECAP-O → ICECAP-A** | |
| --- | --- | --- | --- |
| Stability 4 | Security 4 | Attachment 4 | Attachment 4 |
| Stability 3 | Security 3 | Attachment 3 | Attachment 4/3 |
| Stability 2 | Security 2 | Attachment 2 | Attachment 2 |
| Stability 1 | Security 1 | Attachment 1 | Attachment 1 |
| Attachment 4 | Attachment 4/3 | Security 4 | Stability 4 |
| Attachment 3 | Attachment 3 | Security 3 | Stability 3 |
| Attachment 2 | Attachment 2 | Security 2 | Stability 2 |
| Attachment 1 | Attachment 1 | Security 1 | Stability 1 |
| Autonomy 4 | Control 4 | Role 4 | Achievement 4 |
| Autonomy 3 | Control 3 | Role 3 | Achievement 3 |
| Autonomy 2 | Control 2 | Role 2 | Achievement 2 |
| Autonomy 1 | Control 1 | Role 1 | Achievement 1 |
| Achievement 4 | Role 4 | Enjoyment 4 | Enjoyment 4 |
| Achievement 3 | Role 3 | Enjoyment 3 | Enjoyment 4/3 |
| Achievement 2 | Role 2 | Enjoyment 2 | Enjoyment 2 |
| Achievement 1 | Role 1 | Enjoyment 1 | Enjoyment 1 |
| Enjoyment 4 | Enjoyment 4/3 | Control 4 | Role 4 |
| Enjoyment 3 | Enjoyment 3 | Control 3 | Role 3 |
| Enjoyment 2 | Enjoyment 2 | Control 2 | Role 2 |
| Enjoyment 1 | Enjoyment 1 | Control 1 | Role 1 |

Capability attribute levels, 4 – top (full) capability, 1 – bottom (no) capability for both ICECAP-A and ICECAP-O
